# Supplementary material for: Natural hybridization in heliconiine butterflies: the species boundary as a continuum
Source: BMC Evol Biol. 2007 Feb 23;7:28. doi: 10.1186/1471-2148-7-28 (PMC1821009; doi:10.1186/1471-2148-7-28)
Supplement: Additional File 1 — Hybrids between species of Heliconius and Eueides butterflies: a database. HTML file linking to database of all known wild-caught interspecific hybrid specimens in the Heliconiina, consisting of introductory text, a list of specimens, together with collection data and photographs of the specimens, and links to information about some artificial hybrids and mutants in the group. This is an edited copy of our online database of Heliconius hybrids [102]. To view database, download zip file and extract to a separate folder, then open index.html within that folder. [file 1471-2148-7-28-S1.zip › melcyd12.html]

hybrid melcyd12


---


Hybrid between *Heliconius melpomene {euryas}* and *H. cydno cydno*
Colombia
© J.F. Le Crom / M. Linares

Return to table
of hybrids

To next hybrid
  
To previous hybrid

```
NOTES

No:                      42
Genus of species 1:      Heliconius
Species 1:               melpomene
Subspecies of species 1: {euryas}
Genus of species 2:      Heliconius
Species 2:               cydno
Subspecies of species 2: cydno
Sex:                     m
Country:                 Colombia
Locality:                Caldas: Victoria
Year:                    1966
Photo no.:               melcyd12
Named hybrid:            nr. wernickei
Collection:              Schmidt-Mumm/Instituto Alexander von Humboldt
Collector:               E. Schmidt-Mumm
Author/publication:      Salazar 1993:3
Notes:                   BC->cydno
```

**Last updated:** 7 June 2006

---
